# The Alteration of Brain Interstitial Fluid Drainage with Myelination Development

**DOI:** 10.14336/AD.2021.0305

**Published:** 2021-10-01

**Authors:** Rui Wang, Hongbin Han, Kuangyu Shi, Ian Leigh Alberts, Axel Rominger, Chenlong Yang, Junhao Yan, Dehua Cui, Yun Peng, Qingyuan He, Yajuan Gao, Jingge Lian, Shuangfeng Yang, Huipo Liu, Jun Yang, Chaolan Wong, Xunbin Wei, Feng Yin, Yanxing Jia, Huaiyu Tong, Bo Liu, Jianbo Lei

**Affiliations:** ^1^Department of Radiology, Peking University Third Hospital, Beijing, China.; ^2^Beijing Key Lab of Magnetic Resonance Imaging Device and Technique, Beijing, China.; ^3^Institute of Medical Technology, Peking University Health Science Center, Beijing, China.; ^4^Department of Nuclear Medicine, University of Bern, Switzerland.; ^5^Department of Informatics, Technical University of Munich, Garching, Germany.; ^6^Department of Neurosurgery, Peking University Third Hospital, Beijing, China.; ^7^Department of Radiology, Beijing Children’s Hospital, Capital Medical University, National Center for Children’s Health, Beijing, China.; ^8^Key Laboratory of Natural and Biomimetic Drugs, School of Pharmaceutical Sciences, Peking University, Beijing, China.; ^9^Biomedical Engineering Department, Peking University, Beijing, China.; ^10^Department of Neurosurgery, Aerospace Center Hospital, Beijing, China.; ^11^Department of Neurosurgery, PLA General Hospital, Beijing, China.; ^12^Institute of Applied Physics and Computational Mathematics, Beijing, China.

**Keywords:** brain extracellular space, interstitial fluid, tracer-based MRI, brain development

## Abstract

The integrity of myelination is crucial for maintaining brain interstitial fluid (ISF) drainage in adults; however, the mechanism of ISF drainage with immature myelin in the developing brain remains unknown. In the present study, the ISF drainage from the caudate nucleus (Cn) to the ipsilateral cortex was studied at different developmental stages of the rat brain (P 10, 20, 30, 40, 60, 80, 10-80). The results show that the traced ISF drained to the cortex from Cn and to the thalamus in an opposite direction before P30. From P40, we found impeded drainage to the thalamus due to myelin maturation. This altered drainage was accompanied by enhanced cognitive and social functions, which were consistent with those in the adult rats. A significant difference in diffusion parameters was also demonstrated between the extracellular space (ECS) before and after P30. The present study revealed the alteration of ISF drainage regulated by myelin at different stages during development, indicating that a regional ISF homeostasis may be essential for mature psychological and cognitive functions.

The extracellular space (ECS) is essential to normal brain function, and during normal brain development it undergoes significant change [1-5]. Recent studies have verified the drainage route of the brain interstitial fluid (ISF) from the deep caudate nucleus (Cn) to the ipsilateral cortex along the matured myelin fibers in adult rats [6,7]. Disturbed ISF drainage was found in the cuprizone-induced demyelinated and aggressive C6 glioma models [7]. These results indicate that myelin integrity determines the normal drainage of ISF within brain ECS [5-7]. This discovery of a new role for myelin in regulating ISF draining may provide new perspectives for understanding the underlying mechanism of demyelination-related brain disorders [6]. It has long been known that, in humans, myelination maturation continues into the post-natal period [7] and myelination of the peripheral nervous system continues into childhood [8]. The finding that the integrity of myelin has a role in ISF drainage in adult rats implies that myelination of the immature brain might have a potential role in influencing or regulating ISF drainage in the developing brain.

In the present study, a novel method of tracer-based magnetic resonance imaging (MRI) with ECS diffusion mapping (D_ECS_-Mapping) technique [10,11] was used to study the ISF drainage at different post-natal developmental stages in rat brains (postnatal days [P] 10, 20, 30, 40, 60, 80, P10-80). Dynamic changes in ECS structure and ISF drainage *in vivo* were detected and compared. Morphological changes were observed by laser scanning confocal microscopy (LSCM) and electron microscopy (EM). Cognitive, social, motor, and sensory function during development were assessed by various behavior tests. The significance of brain ECS and ISF on brain development and the effect on diseases with dysmyelination and demyelination were discussed.

## MATERIALS AND METHODS

### Experimental animals

This study was approved by the Ethics Committee of the Peking University Health Science Center (LA 2012-016). All experimental procedures and protocols involving animals in this study were performed following the Chinese guidelines for the use of experimental animals. The male Sprague-Dawley rats were stratified into the juvenile (P10, P20, P30, and P40) and adult (P60 and P80) age groups (n = 18 in each group with each experiment). All rats were housed under a 12-h light/dark cycle under controlled temperature (22 ± 1°C) and humidity (60 ± 5%).

### Stereotactic injection

The Gd-DTPA tracer (Magnevist; Bayer Schering Pharma AG, Berlin, Germany) and the optic-magnetic bimodal molecular tracer Gd-DO3A-ethylthiouret-fluorescein (Gd-DO3A-EA-FITC; synthesized by our lab) were diluted to 10 mmol/L [6, 13, 14]. The tracers were delivered for 10 min with an automated drug administration system (Harvard Apparatus, Holliston, MA, USA). The administration was followed by a waiting period of 5 min [6,11]. The position of the Cn was localized on the skull surface using the bregma as a reference point. The sites of the Cn were identified at the following locations for the rats of different ages: P10, anterior: 0.2 mm, right lateral: 2.3 mm, vertical: 4 mm; P20, anterior: 0.3 mm, right lateral: 2.3 mm, vertical: 4 mm; P30, anterior: 0.5 mm, right lateral: 2.8 mm, vertical: 4 mm; P40, anterior: 0.8 mm, right lateral: 3 mm, vertical: 4 mm; P60, anterior: 1 mm, right lateral: 3.5 mm, vertical: 5 mm; and P80, anterior: 1 mm, right lateral: 3.5 mm, vertical: 5 mm. The total volume of the tracer delivered was adjusted according to the brain volume of the rats: P10, 1.2 µl; P20, 1.4 µl; P30, 1.6 µl; P40, 1.8 µl; and P60 and P80, 2 µl.

### Tracer-based MRI and D_ECS_-mapping

After the tracer was injected into the deep brain, imaging of the ISF drainage route was performed with MRI, and all parameters concerning the structure of and molecular diffusion within the ECS could be calculated and presented with the D_ECS_-mapping technique, version 3.1 (MRI Lab, Beijing, China) [10]. This technique was used for dynamic measurements of the ECS structural and functional parameters, including volume fraction (α), tortuosity (λ), diffusion coefficient (D*), clearance coefficient (k’), and half-life (T_1/2_), at the workstation. By using D_ECS_-mapping, the scope and rate of diffusion in the Cn were also determined.

### Laser scanning confocal microscopy (LSCM) ISF tracer imaging

Lucifer yellow CH (Sigma, St. Louis, MO, USA) was selected from a series of fluorescent tracers [5,6], including dextran-tetramethylrhodamine (Invitrogen, Carlsbad, CA, USA), acridine orange (Sigma), and Gd-DO3A-ethylthiourea-FITC, for *in vivo* measurements. The Leica TCS SP8 MP FLIM new merging function can store m × n single images during scanning. The whole image of the sample of a designated area can then be obtained by image reconstruction after scanning. Experimental fluorescence images were collected with the LSCM Merging function [6,9].

### Myelin imaging based on light reflection

Imaging of the myelinated nerve fibers in the rat brain slices was performed using LSCM [12]. Specifically, the reflected laser light was used to image the myelinated nerve fibers. A water-immersion objective (Leica; magnification, 25×; numerical aperture, 1.2) and a 30/70 partially reflective mirror were used for myelin imaging. Lasers with wavelengths of 458, 476, 488, 514, 561, and 633 nm were transmitted through an acousto-optical tunable filter and thus used as excitation light sources. The reflected light was collected by 5-nm receiver channels from three excitation light sources centered around 486-491 nm, 559-564 nm, and 631-636 nm. Each channel was scanned using the xyz scanning mode. Specifically, the light reflected at 561 nm was shown as red, and that at 633 nm was shown as purple. The scanned images were merged into a complete image using the merge function. Post-processing image analysis was performed using the LAS X software.

### Electron microscopy

Electron microscopy was performed using a Nikon2000 transmission electron microscope [6,13,15]. The thickness of the myelin sheath (MSTH) and maturity of myelin in the Cn and internal capsule were measured. The equivalent diameter (ED) and measurement diameter (MD) were used as the parameters of MSTH. The G-ratio represents the maturity of the myelin. The procedures for specimen preparation were performed as described previously.

### Morris water maze

On the day of the behavioral experiments, each animal was placed in the water maze for 1 min to help the rat adapt to the experimental environment and prevent stress during the subsequent trials [16]. For the acquisition trials, each animal underwent four trials per day for five consecutive days. In each trial, the animal was randomly placed at one of four starting positions facing the pool wall. Each trial ended when the animal escaped the maze by landing and staying on a submerged, hidden goal platform for 3?s. Any animal that had not located the platform within 60?s was guided to the platform by hand and allowed to remain in place for 10?s. The rat’s average time to find the platform (escape latency), numbers of passing crossing, and the swim path of each rat were recorded. After 5 days of acquisition trials, the goal platform was removed, and a spatial probe trial was conducted: the mean time spent in the target quadrant, the mean number of times the animals crossed the platform, and their swim paths were recorded. We started the training 3 days prior, then tested and analyzed for 6 days. Thus, the medium ages of the rats were 20, 30, 40, 60 and 80 days after birth.

### T-maze

The test was recorded with a video camera positioned above the maze [17]. On the day of testing, the rat was placed onto the start arm (i.e., the stem of T-maze) such that the rat was facing the stem wall. When the animal entered one of the two other arms, the rat was removed and placed back into the start arm. If an animal did not turn either within the starting arm or into one of the other two arms in 150 s, the trial was terminated, and the animal was taken out and placed back into the start arm. After one rat finished the trials and before another rat was tested, the maze was thoroughly cleaned using an acetic acid solution (0.1%) and carefully dried.

### Barnes maze

The rats underwent training to complete the Barnes maze for 5 days [18]. In the pre-training trial, the rat was placed in the middle of the maze and trained to enter the escape box: the rat was guided to the escape box, where it remained for 2 min. Following the pre-training trial, the first trial was performed. At the beginning of each trial, the rat was placed in the same start location, and the rat was free to explore the maze. The trial ended when the rat entered the goal tunnel or failed to find the goal tunnel after having freely explored the maze for more than 3 min. Rats were tested for four trials per day for 4 days. The trials on the same day were separated by 15 min. After each trial, the entire maze was cleaned, and the maze was rotated to preclude the rat’s use of intra-maze cues. Latency and error times before the goal tunnel was found were recorded.

### Three-chambered social test

The 3-chambered social test was performed to determine the rat’s social function after it was familiar with its environment and after it was enclosed in a metal cage within an experimental box containing another unfamiliar rat. Briefly, the rats were placed into a three-chambered box for a 5-min adaptation period before the test was performed. On the next day, the rats were placed in the middle chamber of the box for 10?min with a shackled, unfamiliar rat in the room on one side and colored toys in the room on the other side. To evaluate the social communication skills of the rat, the number of interactions, and the time spent close to the unfamiliar rat or toys were recorded with ANY-Maze Video Tracking System. After the end of each test, the box and objects were cleaned with 70% ethanol.

### Limb placement test

A limb placement test was used to assess the sensory capabilities of the rats [19], including (a) proprioception, (b) whisker tactile, and (c) visual forward. The tasks were scored in the following manner: ‘proprioception’ was defined as stepping up with the forelimb and hindlimb onto the table after pulling down the forelimb and hindlimb below the level of the table; ‘whisker tactile’, as the stretch of the forelimb after a stimulus was applied to the rat’s whiskers while an examiner held the rat’s trunk; ‘visual forward’, as forelimb flexion while the rat’s tail was held up. The stretch of the forelimbs toward the table was evaluated and scored. All the tasks were observed in triplicate for each animal. The following scoring method was used: normal lifting = 0-point, time of hesitation <2s = 1 point, ≥2s = 2 points. Behavior tests were conducted before the invasive tests.

**Figure 1.**
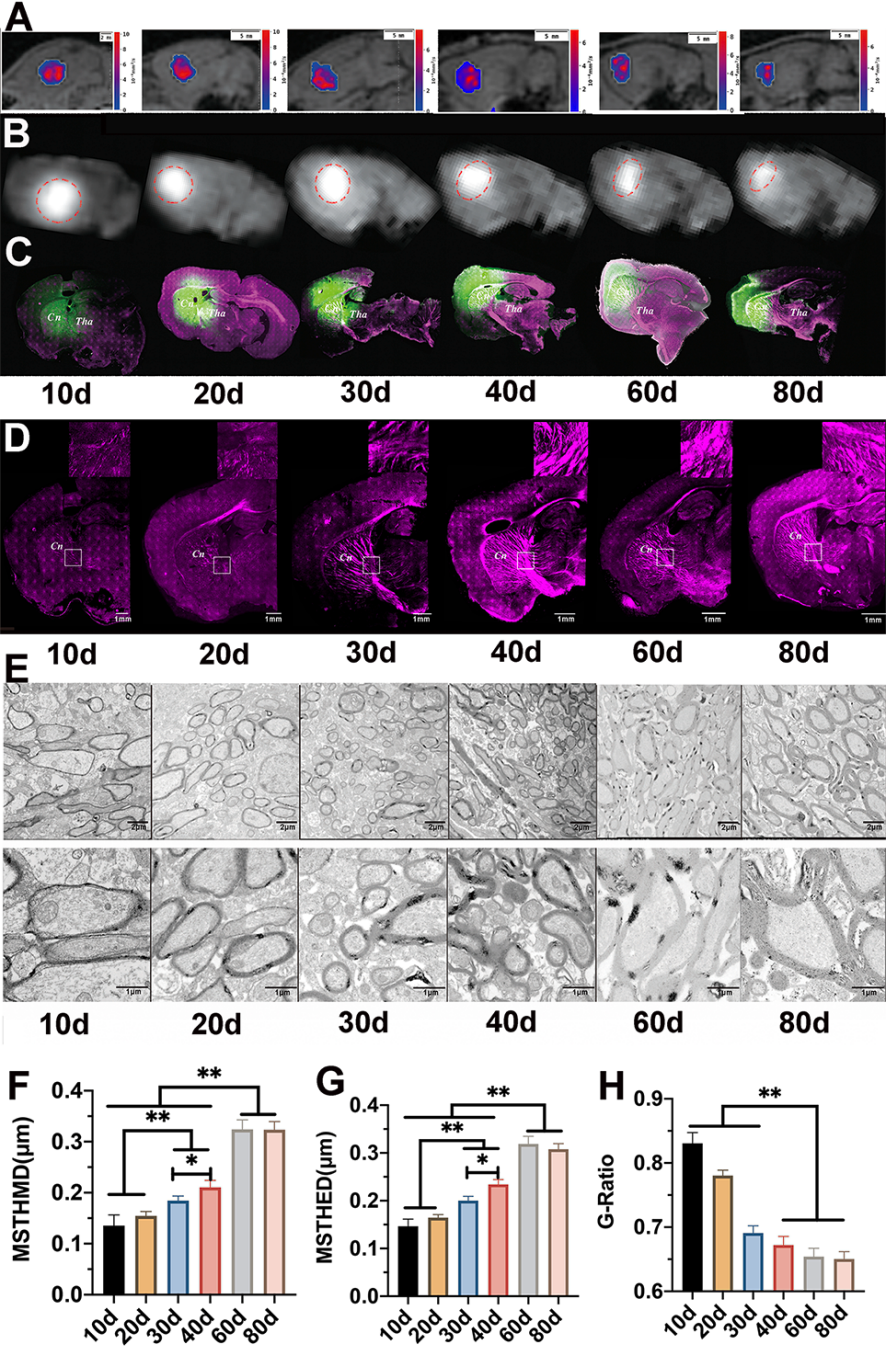
Brain interstitial fluid (ISF) drainage at different ages was observed with the development of myelination. (A) The results of extracellular space diffusion mapping (DECS-mapping) show that, between P10 and P30, the ISF in the caudate nucleus diffuses to larger areas and communicates with the thalamus. After P40, the ISF in the caudate nucleus only drains towards the cortex and does not communicate with the thalamus. (B) Results of tracer-based magnetic resonance imaging (MRI) are the same as DECS-mapping. (C) The same results were confirmed by laser scanning confocal microscopy (LSCM). (D) The results of confocal microscopy show that the barrier structure is composed of compact myelin. (E) Electron microscopy (EM) was used to examine the myelin structure in the barrier region, showing that the myelin thickness could reach 0.24 μm when the barrier is formed on P40 (magnification of the top panel: 1700×; magnification of the bottom panel: 5000×). (F) Equivalent diameter (ED) and (G) measurement diameter (MD) of the thickness of the myelin sheath (MSTH) increased gradually with age. The MSTH of adult rats is thicker than that of the juvenile rats (*p* < 0.01). (H) G-ratio of the myelin decreases gradually with age and stabilizes after P40 (*p* > 0.05).

**Figure 2.**
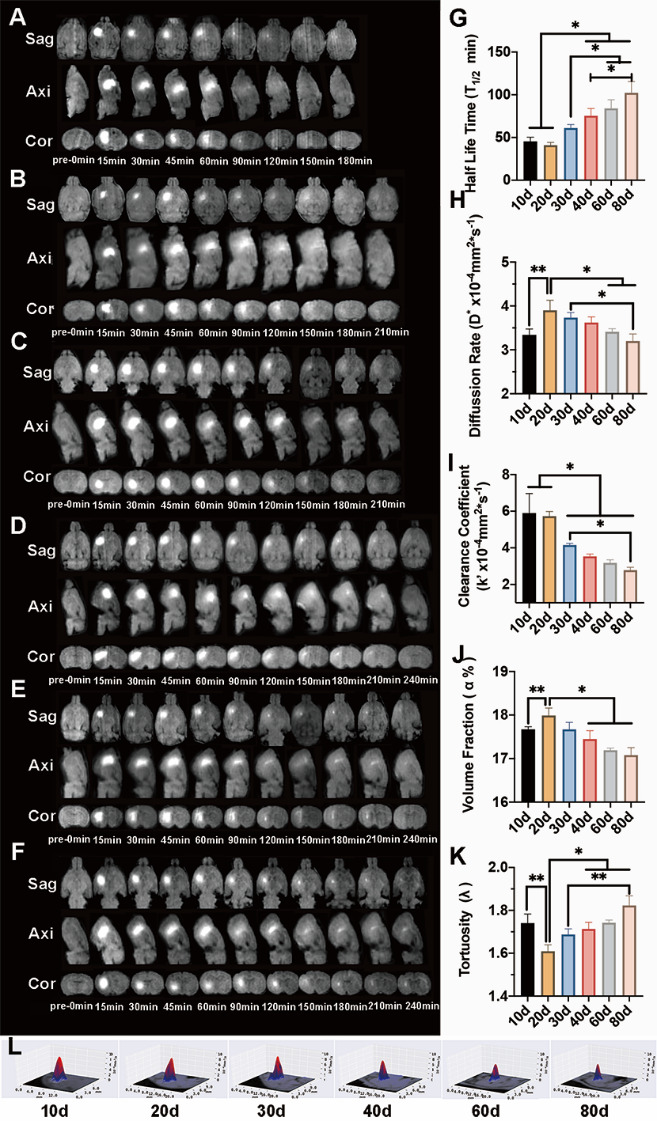
Changes in the brain extracellular space (ECS) structure and interstitial fluid (ISF) drainage in rats during development. (A) Tracer-based magnetic resonance imaging (MRI) in P10 rats shows the complete elimination of the tracer 160 min after injection; (B) Tracer-based MRI in P20 rats shows the complete elimination of the tracer 180 min after injection; (C) Tracer-based MRI in P30 rats shows the complete elimination of the tracer 210 min after injection; (D) Tracer-based MRI in P40 rats shows the complete elimination of the tracer 240 min after injection; (E) Tracer-based MRI in P60 rats shows the complete elimination of the tracer 240 min after injection; (F) Tracer-based MRI in P80 rats shows the complete elimination of the tracer 240 min after injection. (G) The half-life of the tracer (T_1/2_) decreased gradually with age and significantly differed between each group (*p* < 0.05). (H) Diffusion rate (D*) initially increased (P10 vs P20, p < 0.01), later decreased, and finally became stable after P40 (*p* > 0.05). (I) The clearance coefficient (k’) of ISF also decreased with age but did not differ between the P40 and adult rats. (J) The volume fraction (α) initially increased (P10 vs P20, *p* < 0.01), decreased from P20 to P30, and became stable at P30, after which it did not change significantly (*p* > 0.05). (K) Tortuosity (λ) initially decreased (P10 vs P20, *p* < 0.01) and then increased. (L) DECS-mapping was performed to examine the distribution rate and scope of ISF in Cn. The image shows that D* decreased from P40.

**Table 1:** Pearson’s correlation coefficient (r) and p-values pertaining to the relationships between thickness, G-ratio of myelin, and the parameters of ECS structure and ISF drainage.

Variable	k’		T_1/2_		D		α		λ	
	r	p	r	p	r	p	r	p	r	p
MSTHMD	-0.709^**^	0.000	-0.625^**^	0.001	-0.220	0.234	-0.161	0.404	0.332	0.098
MSTHED	-0.729^**^	0.000	-0.653^**^	0.001	-0.185	0.320	-0.178	0.356	0.311	0.122
G-ratio	0.799^**^	0.000	0.498^*^	0.016	0.057	0.761	0.099	0.610	-0.140	0.496

*p<0.05, **p<0.01. Abbreviations: ECS; extracellular space; ISF; interstitial fluid; MSTHED; thickness of the myelin sheath-equivalent diameter; MSTHMD; thickness of the myelin sheath-measurement diameter.

### Statistical analysis

Statistical analysis was performed using SPSS 26.0 (Version 26.0. IBM Corp., Armonk, NY, USA). Data that followed a normal distribution were expressed as mean ± standard error. One-way ANOVA was used to compare the data among groups and *t* test was used to compare the data between two groups. The categorical variables of the rats were analyzed with the Chi-square test. Data that did not follow a normal distribution were log-transformed to follow a normal distribution. Correlations were assessed between two variables using general linear regression analysis. Results were considered statistically significant if *p*-values were <0.05.

## RESULTS

### Dynamic changes of brain ECS and ISF drainage with the development of myelination

Using tracer-based MRI, the traced brain ISF was found to drain from Cn bi-directionally to the ipsilateral cortex and the adjacent thalamus in the P10, P20, and P30 groups. In the P40 and the adult (P60, P80) groups, the ISF drained only to the cortex, and its communication with the thalamus was impeded (Fig. 1A-C). A significant difference in the ISF drainage rate was demonstrated. The T_1/2_ of the traced ISF decreased gradually with age and was significantly different across the age groups (Fig. 2G).

The rat brain myelination processes of different groups were imaged and quantitatively measured by EM. The myelin density and thickness increased gradually with age, and the MSTH of adults was found to be thicker than that of the juveniles (*p*<0.01; Fig. 1E-G; Supplementary Table 1). In addition, the maturity of the myelin improved and fully developed after P40 (G-ratio: P40 vs P60 vs P80, *p* > 0.05; Fig. 1H).

LSCM was also used to further determine the progress of the structural formation of this regional ISF drainage system, reconfirming that the barrier structure was formed by myelin and that the density and thickness of myelin increased significantly with age (Fig. 1D).

The diffusion and clearance rates of ISF were significantly different across the establishment of the barrier at P40. The barrier structure was constructed with compact myelin. Pearson correlation analysis showed that the myelin’s thickness and maturity significantly correlated with the half-life and clearance rate of the ISF (Table 1).

**Table 2:** Pearson’s correlation coefficient (r) and p-values pertaining to the relationships between thickness, G-ratio of myelin and cognitive, motor function of rats.

Variable	Water Morris		Barnes Morris				3-Chambered Social Test			
	Escape latency		Latency		Errors		Interaction time		Interaction numbers	
	r	*p*	r	*p*	r	*p*	r	*p*	r	*p*
MSTHMD	-0.695^**^	0.000	-0.625^**^	0.001	-0.529^**^	0.007	0.396	0.068	0.353	0.091
MSTHED	-0.669^**^	0.001	-0.653^**^	0.001	-0.544^**^	0.005	0.347	0.114	0.337	0.108
G-ratio	0.612^**^	0.002	0.498^*^	0.016	0.763^**^	0.000	-0.462^*^	0.031	-0.441^*^	0.031

*p*<0.05; **p<0.01; Abbreviations: MSTHED: the thickness of the myelin sheath-equivalent diameter; MSTHMD: the thickness of the myelin sheath-measurement diameter.

**Figure 3.**
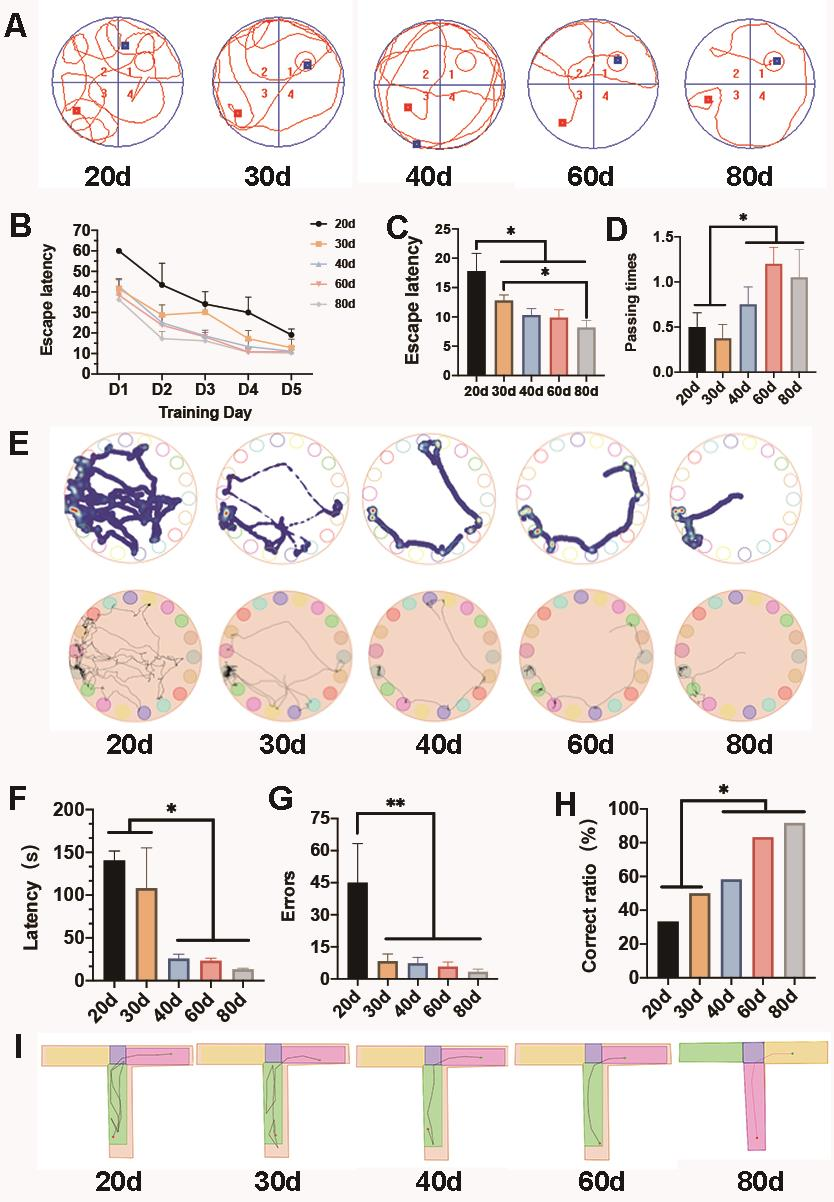
Change in cognitive function during development. (A) Tracks in the Morris water maze tests. (B) The rats were trained for 5 days, and the escape latency of the rats decreased gradually. (C) Escape latency decreased with age. The time spent by adult rats was much shorter than those spent by P20 and P30 rats (*p* < 0.01), while there was no difference between P40 rats and the adults. (D) The adult groups passed more frequently than the P20-30 groups (*p* < 0.01). (E) Tracks in the Barnes maze. (F) The latency of the adult groups was significantly shorter than those of the P20 and P30 groups (*p* < 0.05). There was no difference between the P40 and the adult groups (*p* > 0.05). (G) Error times decreased and were significantly different between the P20 rats and their older counterparts (*p* < 0.05). (H) The correct ratios of the rats were analyzed with the Chi-square test (). The ratios of the P40 and adult rats were much higher than those of the P20 and P30 rats (*p* < 0.05). There was no difference between the P40 and adult groups (*p* > 0.05). (I) Tracks in the T-maze tests.

**Figure 4.**
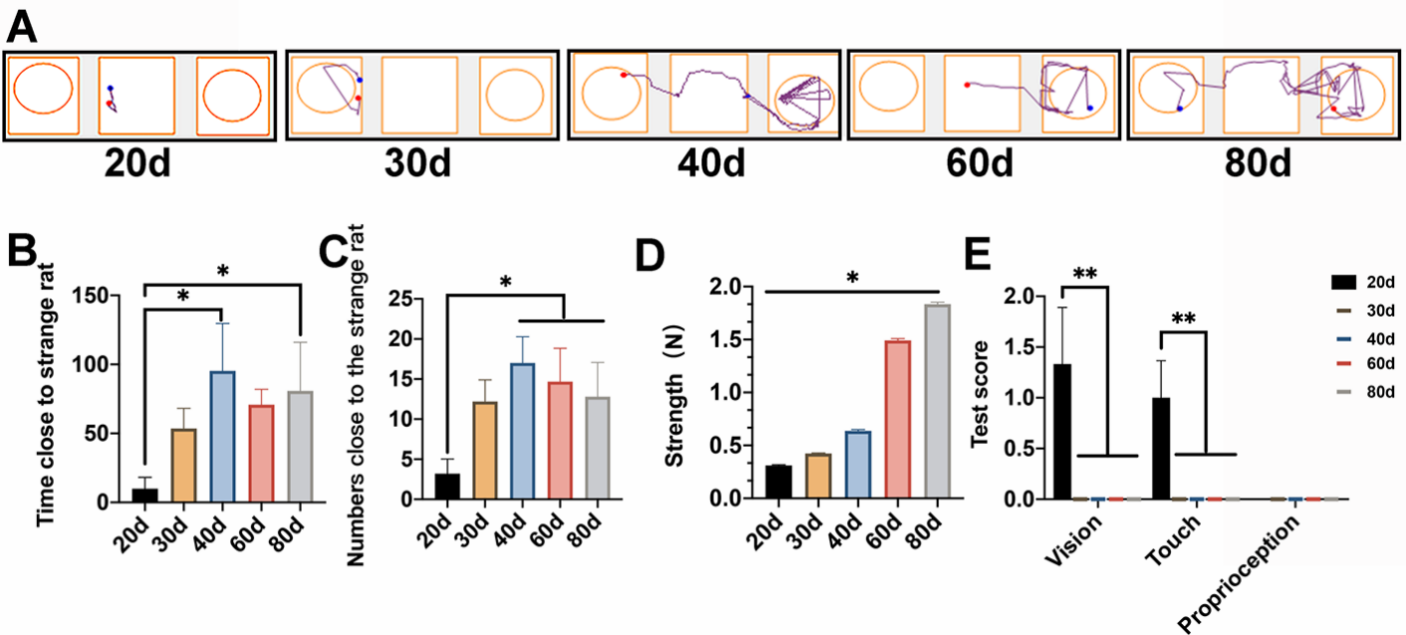
Changes of social, motor, and sensory function during development. (A) Tracks in the three-chambered social tests. (B) The times spent by the P40 and P80 rats were much longer than that of the P20 rats. (C) Entry times of the P40, P60, and P80 groups were greater than that of the P20 group. (D) Angles measured by the inclined plane test did not differ between the P40 and adult groups (*p* >0.05). (E) The limb placement test indicated that the sense of vision and touch had become well developed after P30, while the sense of proprioception was fully developed at P20.

### Measurement of brain ECS tortuosity (λ) and volume fraction (α) under different ISF drainage patterns

The ECS diffusion parameters were measured using the DECS-mapping technique (Fig. 2A-F, L). Both the α and diffusion rate of ECS (D*) initially increased (P10 vs P20, *p* < 0.01), then decreased, and α remained stable after P30 (P30 vs P40 vs P60 vs P80, *p* > 0.05), while D* remained stable after P40 (P40 vs P60 vs P80, *p* > 0.05) (Fig. 2H, J; Supplementary Table 2). The ECS λ initially decreased (P10 vs P20, *p* < 0.01), then increased from P20 to P40 (P20 vs P40 *p*<0.05, P30 vs P80 *p*<0.01), then remained stable thereafter (Fig. 2K). The k’ of ISF significantly decreased from P10 to P40 (*p* < 0.05), and no significant difference was found among P40, P60, and P80 (Fig. 2I; Supplementary Table 2).

### Changes in cognitive function with the alteration of the brain ISF drainage

To investigate learning and memory functions before and after the barrier was established, we conducted several behavioral tests firstly: the Morris water maze, T-maze, and Barnes maze. Three maze tests showed that the rats’ cognitive function gradually developed with age and peaked at P40 and remained stable afterward.

The Morris water maze showed that the time taken to reach the target quadrant declined with age (Fig. 3A, Supplementary Table 3). The latency of the P20 group significantly differed from those of the other groups (P20 vs P30, *p* < 0.01; P20 vs P40 *p* < 0.01; P20 vs P60 *p* < 0.01; P20 vs P80 *p* < 0.01). The latency of the P30 group was significantly longer than that of the P80 group (P30 vs P80, *p* < 0.05; Fig. 3C). The number of platform crossings increased with age. The number of platform crossings was significantly higher in the adult groups than in the P20 and P30 groups (P20 vs P60, *p* < 0.01; P30 vs P60 *p* < 0.01; P30 vs P80 *p* < 0.05). Both the latency and crossing times did not differ between the P40 and adult groups (P40 vs P60 vs P80, *p* > 0.05) (Fig. 3D; Supplementary Table 3). Further analysis showed that the latency was significantly correlated with barrier structure parameters (Table 2).

The results of the Barnes maze showed that the latency declined with age (Fig. 3E; Supplementary Table 4). The latency of the P20 group was significantly longer than those of the P40 and adult groups. (P20 vs P40, *p* < 0.01; P20 vs P60, *p* < 0.01; P20 vs P80, *p* < 0.01). The P30 group’s latency was also significantly longer than that of the adult groups (P30 vs P60, *p* < 0.05; P30 vs P80, *p* < 0.05). No difference in latency was found between the P40 and adult groups (P40 vs P60 vs P80, *p* > 0.05; Fig. 3F). The error times also decreased with age, and significant differences were found in the error times between the P20 and the remaining groups (P20 vs P30, *p* < 0.05; P20 vs P40 *p* < 0.05; P20 vs P60 *p* < 0.05; P20 vs P80 *p* < 0.05; Fig. 3G). The latency was significantly correlated with parameters of the barrier structure (Table 2).

The results of the T-maze test were analyzed with the Chi-square test (χ^2^) (Fig. 3I; Supplementary Table 5). The results showed that the correct ratios of the P40 and adult rats were much higher than those of the P20 and P30 rats (P20 vs P40, P60, P80, *p* < 0.01; P30 vs P40, P60, P80, *p* < 0.05). No significant difference was found between the P40 and adult groups (Fig. 3H, J).

### Changes of the social, motor, and sensory functions with the alteration of the brain ISF drainage

We conducted the 3-chambered social test to assess changes in social functioning across age (Fig. 4A; Supplementary Table 6). The results revealed that the number of entries into and the time stay with the novel rat in the chamber housing increased with age. Time spent by the P40 and P80 rats was much longer than that spent by the P20 rats (Fig. 4B). The P40, P60, and P80 rats entered the chamber with the novel rat more frequently than did the P20 rats (Fig. 4C). Furthermore, we found significant correlations between the interaction time and the maturity of myelin (*r* = -0.441*, *p* < 0.05), as well as the entry numbers and maturity of myelin (*r* = -0.441*, *p* < 0.05) (Table 2).

The rats’ grip strength increased consistently with age and differed significantly between the groups (Fig. 4D; Supplementary Table 7). The limb placement test revealed that the sense of vision and touch had been well developed after P30, while the sense of proprioception had been fully developed at P20 (Fig. 4E; Supplementary Table 8).

**Figure 5.**
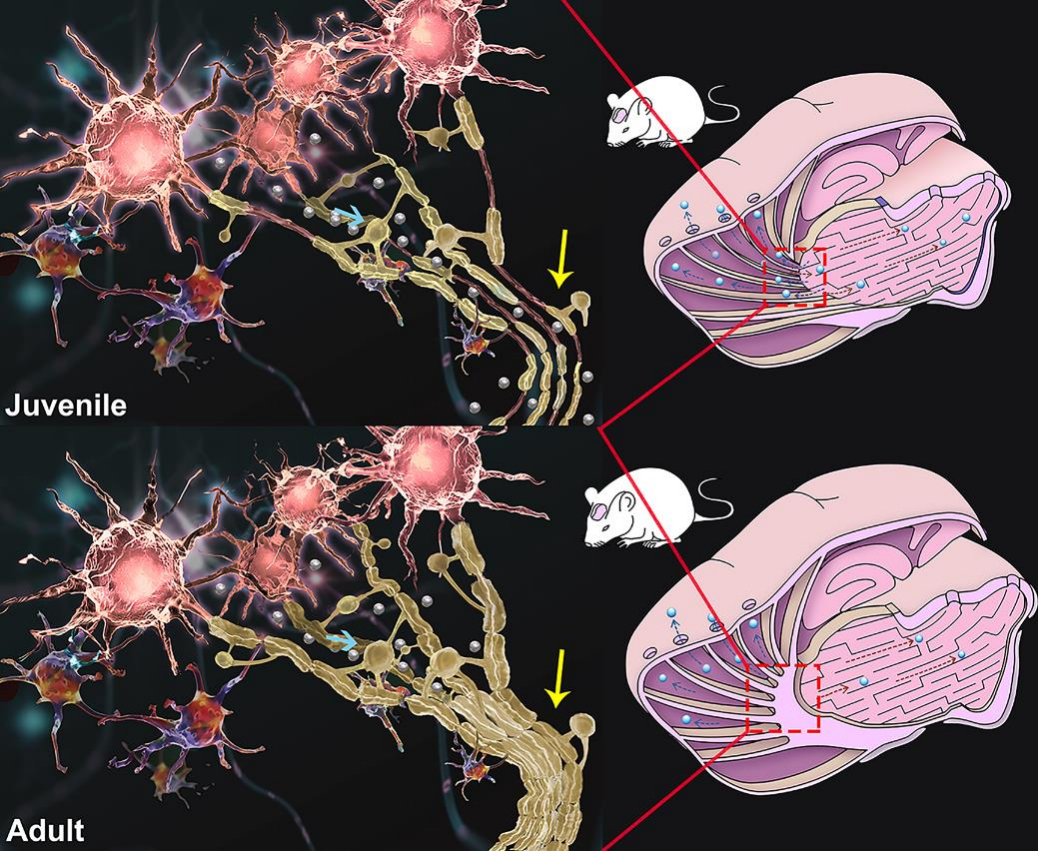
ISF drainage routes in juvenile (≤P30 days) and adult rats. In juvenile rats (≤P30 days), the brain has not been fully myelinated, the oligodendrocytes are small, myelin sheaths are thin, and many axons are nonmyelinated. The interstitial fluid (ISF) was traced from the caudate nucleus (Cn) drained to the ipsilateral cortex along myelin fiber tracts and can communicate with the ISF in the thalamus (Tha) in the opposite direction. After P40, ISF drainage in Cn became regionalized; the movement of ISF to the adjacent thalamus was completely impeded by a barrier structure composed of compact myelin (yellow arrow). The oligodendrocytes in adult rats were more developed than the juvenile rats (blue arrow).

## DISCUSSION

In the present study, the effects of developing myelination on the direction and speed of ISF drainage were clarified. An initially highly connected ECS becomes compartmentalized by day 40 due to the maturation of myelin, which resulted in a more regular one-way ISF drainage from the ECS of Cn to its ipsilateral cortex (Fig. 5). This change was found to be accompanied by an improvement in the rats’ cognitive and social functions. This result suggests that regional homeostasis might have a role in the development of the psychological and cognitive capabilities in adult rat brains.

Neuronal migration and differentiation are of vital importance for brain development [3,20]. The migration of neurons in the human brain continues until approximately 1 year after birth [1,2,20]. It is an extremely complicated process, including precise cellular localization; guidance by various structures, such as the shafts of radial glial cells; and regulation of various proteins [1,21,22]. Moreover, neuronal differentiation is also highly complex and includes the formation of axons and dendrites, the involvement of neurotransmitters, and the formation of cytoskeletons and complex cell membrane structures [1,2,22]. The ECS of the brain is an important medium for cell migration and neurotransmitter transport, and undergoes drastic changes during brain development [5,23]. An asynchronous development was demonstrated between the α of ECS and the brain ISF drainage. The structure of ECS was well developed at P30, while the initial bidirectional drainage of ISF became unidirectional by P40. ECS is surrounded by the cell membranes and its attached extracellular matrix (ECM) [1,5,6,23]. Neural stem and progenitor cells reach their developmental peak before P10, which would squeeze the space and change the molecular motion within ECS [1,3,24]. In the following 10 days, the stem and progenitor cell populations are known to decrease significantly, which leads to an increased α [1]. Peak myelination was observed at P20. An oligodendrocyte gained about three times its weight in the membrane per day [1,3,25,26]. Thus, α of ECS from P10 to P20 continually decreases and reaches the level of the adult brain at P30.

The regulating mechanisms of brain ISF drainage are complex [5,6,27,28]. The ISF originates from capillaries, neural cells, and communicates with CSF in the subarachnoid space [5]. As the main boundary of the brain ECS in the white matter, myelin plays an important role in regulating both the direction and speed of ISF drainage [5,6]. Significantly disturbed ISF drainage has been observed in a demyelinated rat model caused by chemical toxicity [6]. A recent study by Li et al. demonstrated downregulated ISF drainage following neuronal excitation [13]. The study showed that the reduced ISF drainage was caused by the slow diffusion in ECS and the release of excitatory neurotransmitters, including glutamate, glutamine, N-acetylaspartate, and aspartate [13]. Under different anesthesia, the release of noradrenaline (NA) in the brain ECS could also determine the drainage rate of ISF [29]. It has been reported that, before P30, neurotransmitters changed drastically to facilitate the development, differentiation, and migration of neural cells [30,31]. The brain ECS is an irregular, tortuous, and narrow space among capillaries and neural cells, thus the drastic development of neural cells induced by neurotransmitters may have altered the ECS structure, thus changing the ISF drainage. The correspondingly changed ISF drainage follows the drainage pattern of adult rat brain at P40. The traced ISF from the Cn drained to the ipsilateral cortex along myelin fiber tracts, while in the opposite direction, its drainage to the adjacent thalamus ceased. The ECS was thus compartmentalized. In our previous study, we verified that the regionalized ISF drainage is important for maintaining normal brain functions, which can be impeded with cuprizone-induced demyelination [5,6,9]. Therefore, we hypothesized that a regionalized ISF drainage provides local homeostasis of the working environment for neurons and glial cells. The establishment of local homeostasis was accompanied by fully developed cognitive and social behaviors, and motor or sensory capability in rats. According to a predictive development modeling, 40 days in rats is approximately the age of a human child at 6 years [31,33], this critical time point might mark the juncture between preschool and school ages in a human child. At 6 years old, children have developed proper functions of their touch, auditory, and visual senses, and the capacity of comprehending and memory begin to increase significantly [34,35].

Our results suggest that this regionalized ISF drainage system may be of importance for maintaining the brain’s normal function. Combined with previous research, a matured ISF drainage system can prevent transmitters’ interference from different ECS divisions and may assist the remote signal conduction of neurons in the same ECS along the ISF drainage pathway [5,8,9,29]. Our findings are in keeping with developing literature highlighting a previously underappreciated role of myelin in regulating the ISF drainage [8], and we show that changes in ISF drainage correlate with the developmental changes in the brain function of rats. Based on the above findings, we hypothesize that demyelination could also result in abnormal ISF drainage, for example in an aging rat brain, and might contribute to a decline in cognitive function [36]. These findings highlight potential avenues for therapeutic intervention. For example, recent work shows that the molecular motion in ECS and ISF drainage can be manipulated with a red-light physical intervention [37]. These discoveries may shed light on the diagnosis and treatment for diseases of dysmyelination or demyelination.

In this study, it is reconfirmed that well developed myelin can act as a barrier structure for ISF drainage. However, the underlining mechanism has yet to be explored, *e.g*., the study of ISF drainage in a dysplasia myelin or demyelination model will further verify our conclusion.

## Supplementary Materials

The Supplemenantry data can be found online at: www.aginganddisease.org/EN/10.14336/AD.2021.0305.


