# Supplementary material for: The Alteration of Brain Interstitial Fluid Drainage with Myelination Development
Source: Aging Dis. 2021 Oct 1;12(7):1729–40. doi: 10.14336/AD.2021.0305 (PMC8460314; doi:10.14336/AD.2021.0305)
Supplement: Supplementary file 1 [file AD-12-7-1729-s.pdf]

SUPPLEMENTARY DATA

The Alteration of Brain Interstitial Fluid Drainage with Myelination Development

Rui Wang[1,2], Hongbin Han[1,2,3*], Kuangyu Shi[4,5], Ian Leigh Alberts[4], Axel Rominger[4], Chenlong Yang[2,6], Junhao Yan[3], Dehua Cui[2,3], Yun Peng[3,7], Qingyuan He[1,2,3], Yajuan Gao[1,2], Jingge Lian[1,2,3], Shuangfeng Yang[3,7], Huipo Liu[2,12], Jun Yang[2,6], Chaolan Wong[3,8], Xunbin Wei[3,9], Feng Yin[3,10], Yanxing Jia[3,8], Huaiyu Tong[3,11], Bo Liu[3], Jianbo Lei[3]

© 2021. Wang R et al. Published online at http://www.aginganddisease.org/EN/10.14336/AD.2021.0305

SUPPLEMENTARY DATA

Supplementary Table 1. Thickness and maturity of myelin

	10day	20day	30day	40day	60day	80day
MSTHMD（μm）	0.14±0.07	0.15±0.05	0.18±0.05	0.24±0.07	0.32±0.10	0.32±0.09
MSTHED（μm）	0.15±0.05	0.16±0.04	0.20±0.05	0.23±0.05	0.32±0.09	0.31±0.06
G-ratio	0.83±0.06	0.78±0.05	0.69±0.06	0.67±0.07	0.65±0.07	0.65±0.06

Supplement Table 2. Parameters of ECS structure and ISF drainage.

	10day	20day	30day	40day	60day	80day
$T_{1/2}$	45.39±9.78	40.90±7.54	61.07±8.12	75.40±17.00	84.01±22.85	102.15±23.07
α (%)	1.73±0.03	1.80±0.03	1.76±0.03	1.74±0.04	1.73±0.02	1.73±0.02
λ	1.74±0.09	1.61±0.05	1.69±0.05	1.71±0.06	1.74±0.04	1.82±0.08
D ($\times 10^{-4} mm^2/s$)	3.34±0.33	3.90±0.46	3.73±0.23	3.62±0.26	3.41±0.23	3.2±0.278
k ($\times 10^{-4} mm^2/s$)	5.90±2.14	5.73±0.57	4.15±0.20	3.53±0.26	3.18±0.57	2.78±0.28

Supplement Table 3. Morris Maze.

	20day	30day	40day	60day	80day
Escape Latency(s)	17.82±5.23	12.80±2.11	10.32±2.19	9.89±3.27	8.19±2.42
Passing Times	0.50±0.39	0.38±0.38	0.75±0.47	1.20±0.41	1.05±0.69

Supplement Table 4. Barnes Maze.

	20day	30day	40day	60day	80day
Latency(s)	140.56±54.72	108.19±141.40	25.89±19.91	23.42±13.89	13.41±6.98
Error	45.00±40.91	8.33±5.77	7.33±6.71	5.80±4.71	3.33±3.08

Supplement Table 5. T Maze.

	20day	30day	40day	60day	80day
Correct ratio	33.3%	50.0%	58.3%	83.3%	91.7%

Supplement Table 6. 3-Chambered Social Test

	20day	30day	40day	60day	80day
Time close to the strange rat (s)	9.88±20.37	53.55±29.16	95.34±76.69	70.78±22.41	80.84±78.76
Numbers close to the strange rat	3.20±4.09	12.20±6.06	17.00±7.31	14.67±7.23	12.80±9.58

© 2021. Wang R et al. Published online at http://www.aginganddisease.org/EN/10.14336/AD.2021.0305

SUPPLEMENTARY DATA

Supplement Table 7. Grip strength test.

	20day	30day	40day	60day	80day
Grip strength（N）	0.31±0.04	0.42±0.03	0.64±0.07	1.49±0.10	1.83±0.11

Supplement Table 8. Limb-placement test score.

	20day	30day	40day	60day	80day
Touch	1.00±0.89	0	0	0	0
Vision	1.33±1.37	0	0	0	0
Proprioception	0	0	0	0	0

© 2021. Wang R et al. Published online at http://www.aginganddisease.org/EN/10.14336/AD.2021.0305
